# High-Resolution CT Chest Findings in Suspected COVID-19 Pneumonia Patients With Negative Real-Time Polymerase Chain Reaction Assay

**DOI:** 10.7759/cureus.14023

**Published:** 2021-03-21

**Authors:** Shazia Yusuf, Hafsah Ahmad, Romasa Zeb, Uswa Zeb, Ahmed A Zeb

**Affiliations:** 1 Diagnostic Radiology, Capital Hospital, Islamabad, PAK

**Keywords:** covid-19, hrct chest, rt-pcr, ground glass opacities, subpleural atelectatic bands, reverse halo sign, crazy paving

## Abstract

Objective

The study was conducted with the objective of describing High-resolution computed tomography (HRCT) chest findings of clinically suspected COVID-19 patients having a negative real-time polymerase chain reaction (RT-PCR) assay as well as prevalence and distribution of the HRCT chest manifestations consistent with the diagnosis of COVID-19 pneumonia.

Methods

This descriptive cross-sectional study was conducted prospectively on a total of 48 patients with high clinical suspicion for COVID-19 and a negative RT-PCR assay that was presented to the Diagnostic Radiology Department of Capital Hospital, Islamabad from July 2020 to December 2020. These patients were included via non-probability consecutive sampling, had a positive history of contact with a known COVID-19 patient and/or any two of the following signs and symptoms; fever, cough, malaise, body aches, arthralgia, new-onset loss of taste and smell, and dyspnea or oxygen saturation less than 85%. A detailed history was sought after informed consent and all these patients underwent non-contrast HRCT chest scans that were reported by an experienced consultant radiologist. The scans showing positive features for COVID-19 pneumonia were assessed for the nature and distribution of the disease.

Results

Amongst 48 suspects with negative RT-PCR assay, 38 (79.2%) showed ground-glass opacities, a hallmark feature of COVID-19 pneumonia. A total of 22 (57.89%) of these 38 patients had ground-glass opacities with a crazy-paving pattern, nine (23.68%) mixed ground-glass opacities with consolidation, and seven (18.42%) had pure ground-glass opacities. Among these 79.2% suspects, ground-glass opacities were multifocal in 37 (97.37%), bilateral in 35 (92.10%), peripheral in 36 (94.74%), and dorsally located in 32 (81.6%) cases. Subpleural atelectatic bands were seen in 18 (47.36%) of these, bronchovascular markings were prominent in 15 (39.47%), and reverse halo sign was positive in nine (23.68%) cases. Out of the rest of the cases, three were diagnosed as interstitial lung disease, two as chronic lung disease, and one as active pulmonary tuberculosis.

Conclusion

The majority of clinically suspected cases for COVID-19 showed hallmark findings on non-contrast HRCT chest scans in keeping with coronavirus disease regardless of a negative RT-PCR assay.

## Introduction

COVID-19 caused by SARS-CoV-2 is a highly infective disease that has caused a pandemic infecting more than 103 million people in over 200 countries with deaths over two million. More than 75 million people have recovered from the disease worldwide [1,2]. There is no certainty about the spread, signs/symptoms, diagnosis, and clinical management of coronavirus disease so far [3]. Minor symptoms include a slight fever, mild cough, malaise, body aches, arthralgia, myalgia, and new-onset loss of taste and/or smell. Patients with moderate disease present with signs and symptoms of pneumonia being severe cough, dyspnea, and respiratory distress that progress to sepsis, respiratory and multi-organ failure in the grave stage [4,5]. All the patients showing any of the earlier described signs and symptoms with a travel history to a country with the rapid spread of COVID-19 or a contact history with a known COVID-19 patient within the last 14 days are considered highly suspicious [6].

The clinical manifestations of COVID-19 along with the positive nasopharyngeal and/or oropharyngeal real-time reverse transcription polymerase chain reaction (RT-PCR) assay are considered the standard of reference for diagnosing the disease these days [7]. The sensitivity of the RT-PCR assay is approximated to around 60-71% [8]. A high number of false negatives are likely because of ongoing genomic mutations of SARS-CoV-2, technical and operator ee, lack of resources, and prowess needed for this genomic test [6,9].

A high-resolution computed tomography (HRCT) chest is a rapid diagnostic technique for COVID-19 that has an added benefit of determining the severity, complications, and treatment plan for infected patients [10]. HRCT chest findings typical of COVID-19 pneumonia include bilateral multifocal peripheral-based ground-glass opacities [11].

HRCT has a higher sensitivity of approximately 89% and a moderate specificity of up to 68% [12]. Many types of research show HRCT to be one step ahead of RT-PCR as a first-line screening investigation for COVID-19 in emergency and hospital settings owing to its higher sensitivity and propose the idea of using chest CT or HRCT as a standard of reference along with clinical and laboratory evaluation for the diagnosis of COVID-19 [9,13].

In this study, we determine the HRCT findings in patients clinically suspected of COVID-19 pneumonia with a negative RT-PCR assay. We also aim to assess the nature and distribution of HRCT lesions suggestive of COVID-19 pneumonia in these suspected cases.

## Materials and methods

This prospective, cross-sectional descriptive study was conducted at the Imaging unit of the Diagnostic Radiology Department, Capital Hospital, Capital Development Authority (CDA), Islamabad for a duration of six months, from July 2020 to December 2020.

After receiving an approval letter from the ethical committee of the hospital, informed consent and detailed history of signs and symptoms were sought from a total of 48 COVID-19 suspected patients with negative RT-PCR tests. The subjects were selected via non-probability consecutive sampling. These were entitled patients and thus underwent HRCT chest scans free of cost. Some of these patients had a positive history of contact with a known COVID-19 patient and some presented with any two of the following signs/symptoms; fever, cough, malaise, body aches, arthralgia, new-onset loss of taste and smell, dyspnea, or oxygen saturation less than 85%. All known cases of asthma, tuberculosis, lung carcinoma, and lung metastases were excluded from the study. Children less than 14 years, pregnant and lactating mothers were not included in the study on account of high radiation dose. 

A total of 48 patients were scanned in a 64-Slice Toshiba Aquilion CT Scanner (Canon Medical Systems Corporation, Otawara-shi, Tochigi, Japan). Patients were positioned supine with feet first and were scanned in a craniocaudal direction from the apex of lungs to diaphragm without any intravenous contrast agent. Scans were performed in full inspiration by instructing patients to hold their breath for seven to eight seconds. The technical parameters were kept at 120 kVp, 300 mAs, and a rotation time of 0.5 sec. Scanning slice thickness was 2 mm with an intersection interval of 10 mm that was reduced to 0.5 mm after automatic reconstruction. The scanner was properly disinfected after each suspected COVID-19 patient was scanned.

The reconstructed HRCT images were transferred to Vitrea software at the workstation. Coronal, sagittal, and axial views were assessed on lung and mediastinal windows. Initially, the scan was analyzed by a senior postgraduate resident with the scan being distributed into two categories; suggestive and non-suggestive of COVID-19 pneumonia and was reported within the first 24 hours of scanning by a senior consultant radiologist having 19 years of experience. Evaluation of the scan was based on the type, size, and distribution of opacities with associated bronchovascular and fibrotic changes. The ground-glass opacities were classified as with or without consolidations on the basis of the obscuration of vessels. The ground-glass opacities were further divided into with or without a crazy-paving pattern on thickening of intralobular or interlobular septae. The distribution was explained as peripheral or central based on the involvement of outer one-third and inner two-third of lung parenchyma respectively and ventral or dorsal via a presumed central line. The lung parenchymal lesions were also described in terms of laterality and lobar involvement. The associated findings that strengthened the diagnosis of COVID-19 were also noted, these included subpleural atelectatic bands, prominent bronchovascular markings, and reverse halo signs.

Data entry and analysis were done by using IBM SPSS Statistics 23 and HRCT chest findings for suspected patients with distribution and nature of lesions were expressed as frequencies and percentages.

## Results

Out of a total of 48 patients with clinical suspicion of COVID-19 pneumonia and negative RT-PCR swab, 62.5% (n = 30) were males, 37.5% (n = 18) were females. The mean age of the study group was 48.4 years (range = 30-90 years). The median interval between the RT-PCR test and HRCT scan was found to be one day (range = 0-3 days). 97.9% of the total suspected patients presented with fever and body aches, 68.7% with arthralgia or myalgia, 52% with cough, 33.3% with new-onset loss of taste and smell, 20.8% with shortness of breath, and oxygen saturation less than 85% in room air.

**Figure 1:**
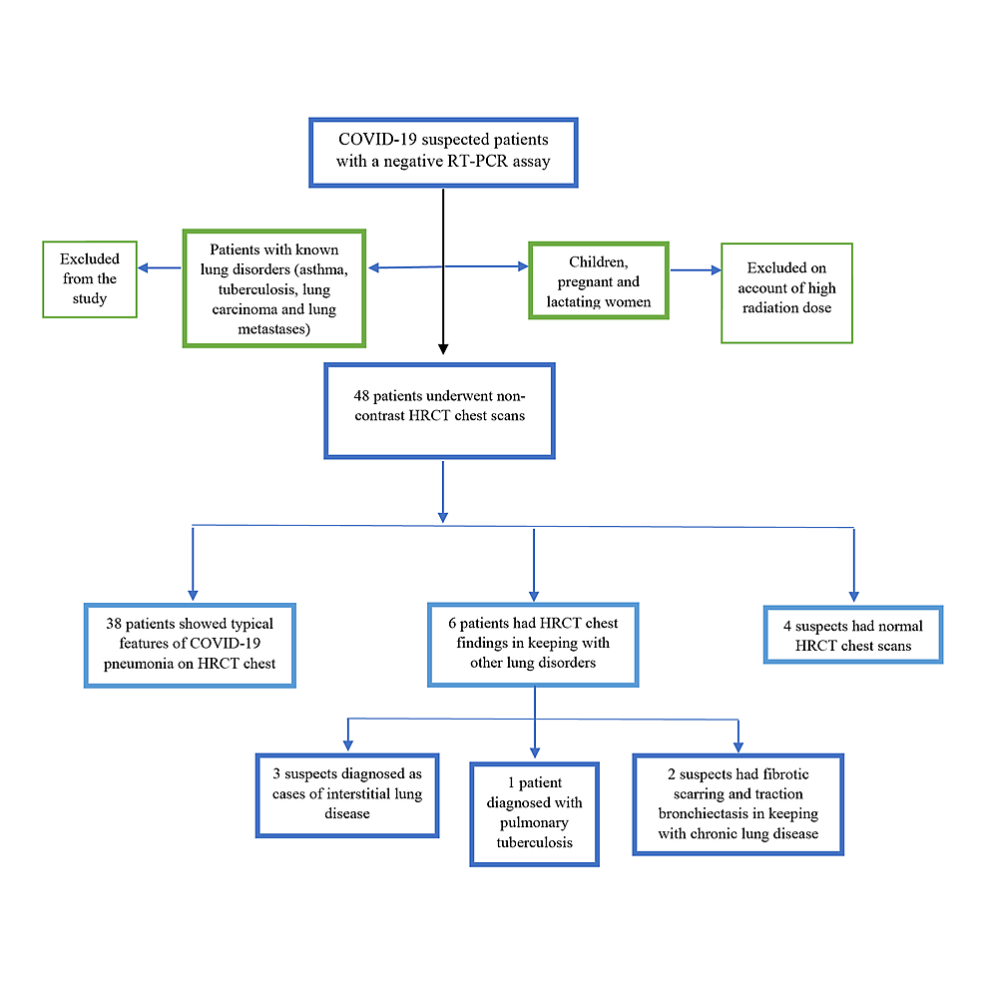
Study flowchart. COVID-19: coronavirus disease 2019; HRCT: High-resolution computed tomography; RT-PCR: Real-time polymerase chain reaction.

Among the total patients, 79.2% (n = 38) had HRCT findings typical of COVID-19, 12.5% (n = 6) had findings suggestive of other diagnoses and 8.3% (n = 4) had normal HRCT chest scans (Figure *1*). The patients with other HRCT findings were diagnosed with different lung disorders (Table *1*). 

**Table 1:** HRCT chest findings suggestive of other diagnoses. HRCT: High-resolution computed tomography.

S. No	Radiological findings	Number of patients	Diagnosis
1	Honeycombing/interstitial thickening	3	Interstitial lung disease
2	Fibrotic scarring	2	Chronic lung disease
3	Bronchiectasis	2	Chronic lung disease
4	Emphysematous lungs with cysts and bullae	2	Chronic lung disease
5	Pleural effusions	1	Tuberculosis
6	Nodules	1	Tuberculosis
7	Cavitary lesions	1	Tuberculosis
8	Lymphadenopathy	1	Tuberculosis

**Figure 2:**
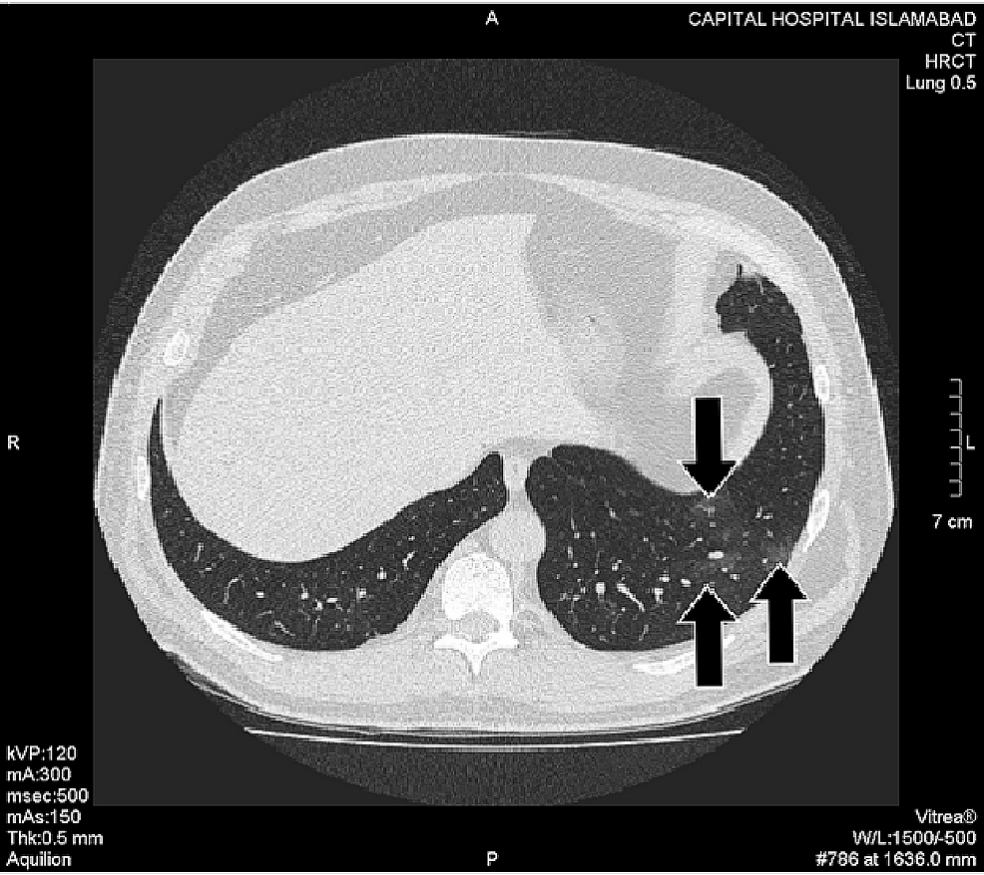
A COVID-19 RT-PCR negative patient with fever and new-onset loss of taste and smell. The axial slice of HRCT scan in lung window shows small patches of pure ground-glass opacities scattered in the peripheral and dorsal distribution in the left lower lobe (black arrows). HRCT: High-resolution computed tomography; RT-PCR: Real-time polymerase chain reaction.

**Figure 3:**
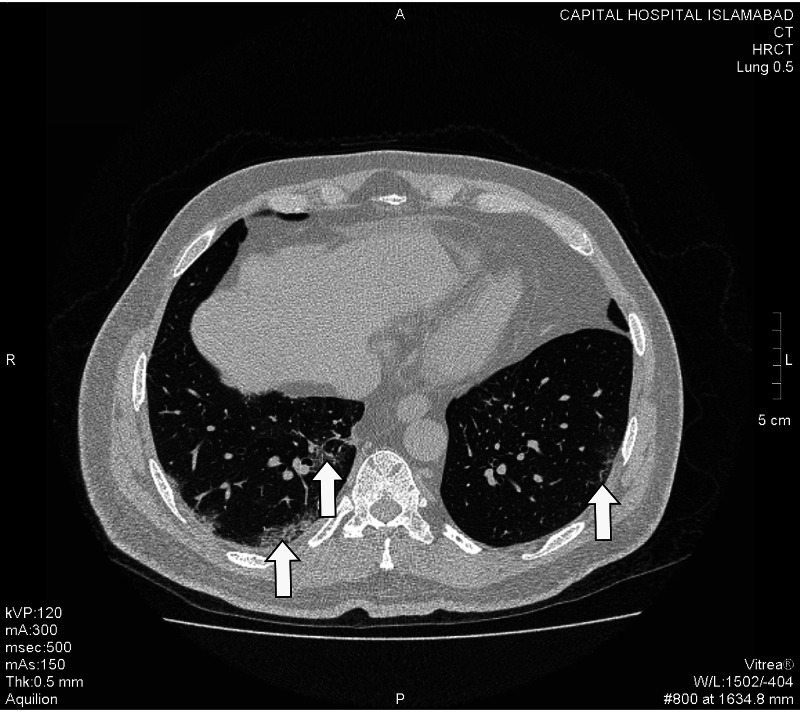
A male patient with cough and shortness of breath. HRCT demonstrates few peripheral and dorsal ground-glass opacities with a crazy-paving pattern in bilateral lower lobes (white arrows). HRCT: High-resolution computed tomography.

**Figure 4:**
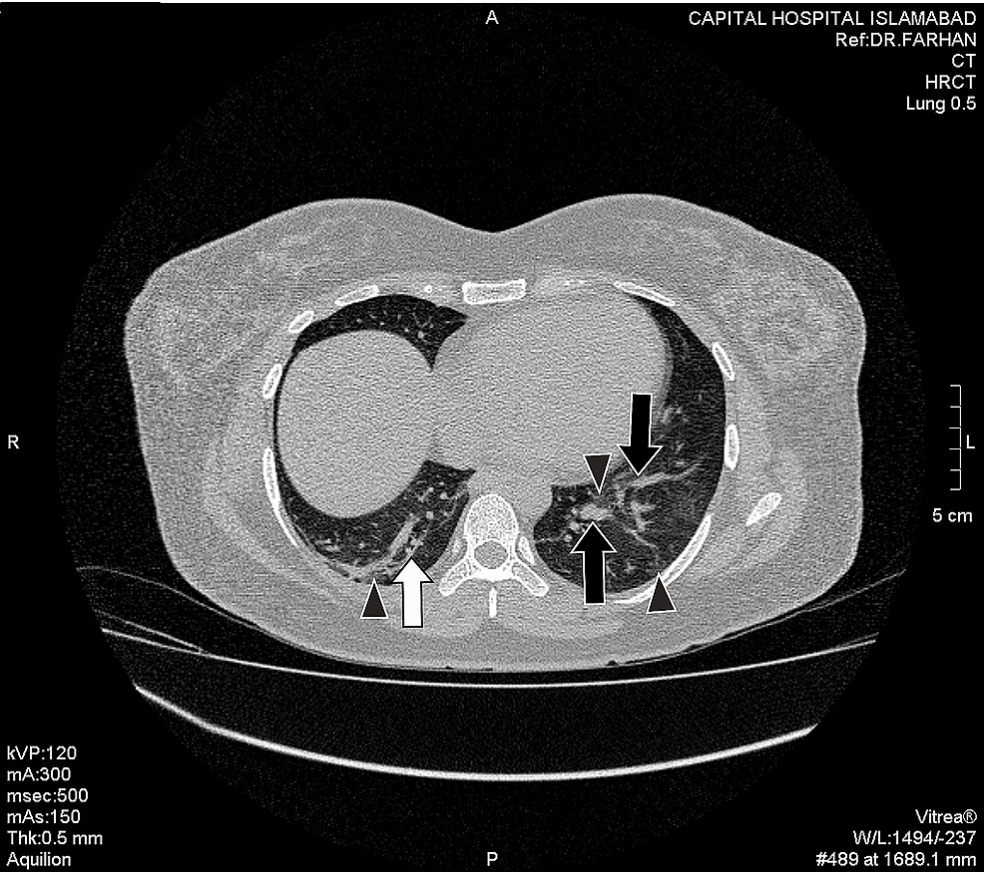
A middle-aged female presented with cough, fever, and body aches on the fourth day of symptoms. HRCT scan illustrates bilateral areas of ground-glass opacities (black arrowheads), the subpleural atelectatic band in right lower lobe (white arrow), and prominent bronchovascular markings in the left lower lobe (black arrows). HRCT: High-resolution computed tomography.

**Figure 5:**
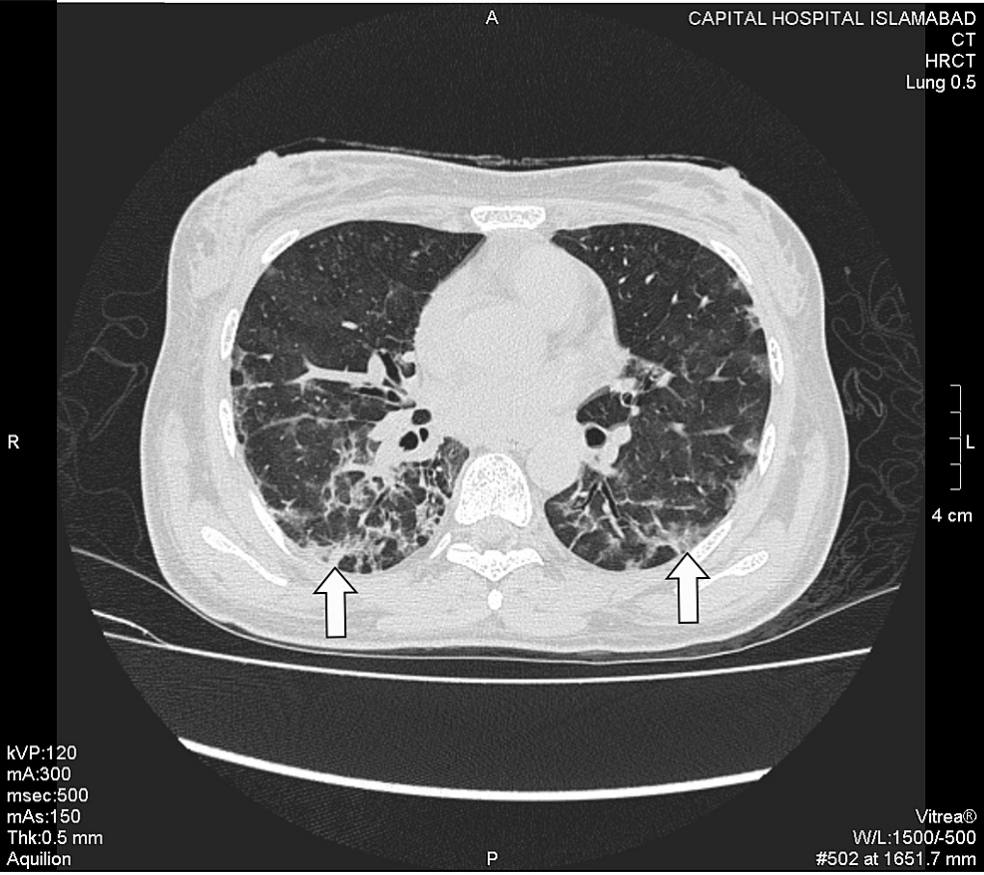
A female patient with severe symptoms. HRCT shows multiple ground-glass opacities mixed with consolidation in bilateral lower lobes (white arrows). HRCT: High-resolution computed tomography.

**Figure 6:**
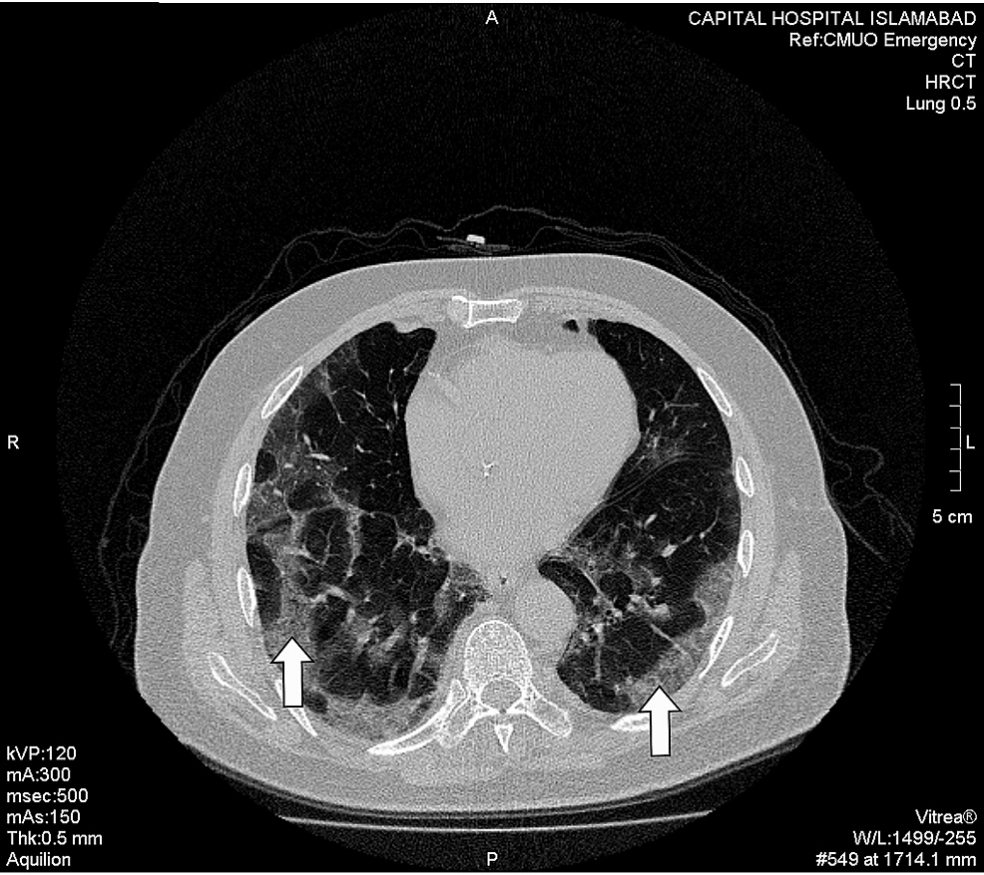
A 78-year-old male patient with shortness of breath and oxygen saturation less than 85% and dropping. HRCT scan exhibits severe disease with multiple predominantly peripheral-based large patches of ground-glass opacities along with a crazy-paving pattern scattered in bilateral lung fields (white arrows). HRCT: High-resolution computed tomography.

Hallmark radiological finding suggestive of COVID-19 pneumonia - ground glass opacification (GGO) was found in 38 patients. These were further categorized as pure GGO, GGO mixed with consolidation, and GGO with a crazy-paving pattern. 57.89% of the patients showed GGO mixed with crazy paving pattern whereas GGO with consolidation and pure GGO were found in 23.68% and 18.42% of the cases, respectively (Figures *2, 3, 4, 5, 6*; Table *2*). The additional findings that supported the diagnosis of COVID-19 were subpleural atelectatic bands, prominent bronchovascular markings, and reverse halo signs. Subpleural atelectatic bands were found in 18 patients and bronchovascular markings were prominent in 15 patients (Figure* 4*; Table *2*). A reverse halo sign was seen in only nine of the 38 patients (Table *2*). 

**Table 2:** HRCT chest features suggestive of COVID-19. HRCT: High-resolution computed tomography.

S. No	Radiological findings	Frequency	Percentage (Out of 38)
1	Ground-glass opacities	7	18.42%
2	Ground-glass opacities with a crazy-paving pattern	22	57.89%
3	Mixed ground-glass opacities and consolidation	9	23.68%
4	Subpleural atelectatic bands	18	47.36%
5	Prominent bronchovascular markings	15	39.47%
6	Reverse halo sign	9	23.68%

Among 38 HRCT scans, 97.37% had evidence of multifocal lesions in contrast to the unifocal lesions (Figures* 2, 3, 4, 5, 6*). Lobar distribution of opacities in lung parenchyma was as follows: right upper lobe 42.10%, right middle lobe 55.2%, right lower lobe 81.6%, left upper lobe 10.5%, and left lower lobe 76.3%. 92.10% of these scans had bilateral and 7.89% had unilateral lesions (Figures *2, 3, 4, 5, 6*). In 94.74% cases, the lesions were in peripheral, 5.26% central, 84.21% dorsal, and 15.79% in ventral distribution respectively (Figures *2, 3, 4, 5, 6*).

## Discussion

Coronavirus disease is a rapidly spreading infection being characterized as a global pandemic [2]. The HRCT chest and RT-PCR assay are both unable to detect COVID-19 infection in the early stages. However, HRCT chest is a rapid method of identifying COVID-19 infected patients compared to RT-PCR assay which requires at least a day or two for the results, and thus, it can play a significant role in the control of the spread of infection especially in the emergency state of the rapid spread of COVID-19 [6]. The inconsistent and low sensitivity of the RT-PCR test indicates that numerous COVID-19 patients cannot be diagnosed leading to a rapid spread in the community [9]. CT chest has been adopted in the diagnostic criteria of COVID-19 for some time being in Hubei, China [13].

Our study determined non-contrast HRCT chest findings in symptomatic COVID-19 cases with an initial negative RT-PCR assay. The time interval between RT-PCR assay and pulmonary HRCT was kept at 0-7 days in the study by Ai et al whereas in our study the time interval was restricted from 0-3 days with a median of one day [14].

According to studies by Xu et al and Ai et al, 31.8% and 75% respectively of RT-PCR negative highly suspected cases had positive findings on HRCT scans characteristically of COVID-19. Our study had similar results to the study done by Ai et al with 79.2% of suspected cases showing HRCT features suggestive of the infection [13,14].

A study done in London showed 20% of lesions to be pure ground-glass opacities and 80% of lesions to be mixed GGO and consolidation whereas our study indicated that 18.42% of ground-glass opacities were pure and only 23.68% were mixed with patches of consolidation. This study showed crazy paving in no patients with subpleural fibrotic lines in 60% of the cases compared to our study which showed crazy paving in 57.89% and subpleural atelectatic bands in 47.36% of the cases. The same study demonstrated the distribution of ground-glass opacities to be bilateral in 100%, peripheral and dorsal in 80% of the cases. In comparison, our study indicated 92.10% bilateral, 94.74% peripheral, and 84.21% dorsal ground-glass opacities. Lower lobes were involved in 100% of the cases with ground glass opacities in this study, on the other hand, our research demonstrated that 81.6% of patients with positive HRCT features had involvement of lower lobes. The same study manifested the ground glass opacities to be predominantly multifocal in nature. Similar to this, our study had 97.37% cases with multifocal lesions [15].

Non-contrast HRCT chest is superior to many other investigations being non-invasive to the patient. However, there are a few limitations to this procedure like its expense and high radiation exposure. The patient has to be shifted to CT scanners to perform an HRCT scan which is difficult for severely ill patients. GGO, the hallmark of COVID 19 on HRCT chest has a wide range of differential diagnoses and the diagnosis of the infection based on HRCT scan findings is subjective depending on the expertise of the radiologists varying from person to person.

However, this research showed that a significant number of COVID-19 pneumonia suspects with negative RT-PCR test had positive chest HRCT features consistent with the diagnosis of coronavirus disease which might be true positive owing to the less sensitive nature of RT-PCR assay. We recommend the use of an HRCT scan along with detailed history and clinical evaluation in patients with diagnostic uncertainty yet high clinical suspicion for COVID-19 infection. We also suggest future studies to be conducted to establish the accuracy of HRCT chest and its inclusion in the diagnostic regimen of patients with a high index of suspicion for coronavirus infection.

## Conclusions

In the light of our conducted research, we can ascertain that the HRCT chest has paramount importance in suspected COVID-19 cases with diagnostic uncertainty. An initial negative RT-PCR test does not rule out the disease, particularly in highly suspicious patients. The only solution to manage the rapid spread of coronavirus disease is early identification and quarantine of COVID-19 patients, hence clinically doubtful COVID-19 cases showing positive features of the infection on the HRCT chest should be treated and isolated as positive cases despite being PCR negative.
